# Advances in Terahertz Biophysics and Chemistry

**DOI:** 10.34133/research.1028

**Published:** 2025-12-22

**Authors:** Zhi Zhu, Tianqi Wang, Huayan Yang, Junquan Zhu, Joseph S. Francisco, Chunlei Wang

**Affiliations:** ^1^ School of Optical-Electrical and Computer Engineering, University of Shanghai for Science and Technology, Shanghai 200093, China.; ^2^ Department of Mechanical Engineering, The Hong Kong Polytechnic University, Hong Kong 999077, China.; ^3^ Shanghai Applied Radiation Institute, School of Environmental and Chemical Engineering, Shanghai University, Shanghai 200444, China.; ^4^ Department of Earth and Environment Science, University of Pennsylvania, Philadelphia, PA 19104-6243, USA.; ^5^ International Joint Laboratory of Catalytic Chemistry, College of Sciences, Shanghai University, Shanghai 200444, China.

## Abstract

As terahertz (THz) science and technology continue to advance, THz biophysics and chemistry have emerged as a pivotal interface, linking molecular-scale insights with transformative innovations in biological, physical, and chemical sciences. This review elucidates the fundamental mechanisms underlying THz–matter interactions, emphasizing the relationship between THz wave physics and molecular motions. We highlight molecular-level effects of THz radiation on water, ion channels, DNA, and proteins, and summarize its biological impacts spanning molecular, cellular, and neurological scales. These mechanistic insights underpin diverse applications, including the control of water transport and molecular dynamics, diagnostics, neurobiology, and emerging therapeutic strategies. Furthermore, we examine advanced methods for detection and characterization in THz biophysics and chemistry, including THz spectroscopy, imaging, and sensing, which bridge fundamental principles with practical implementations, positioning THz technology as a versatile platform for probing and manipulating complex biological and chemical systems. By integrating these recent advances, this review aims to catalyze further research in this rapidly evolving field.

## Introduction

**Fig. 1.**
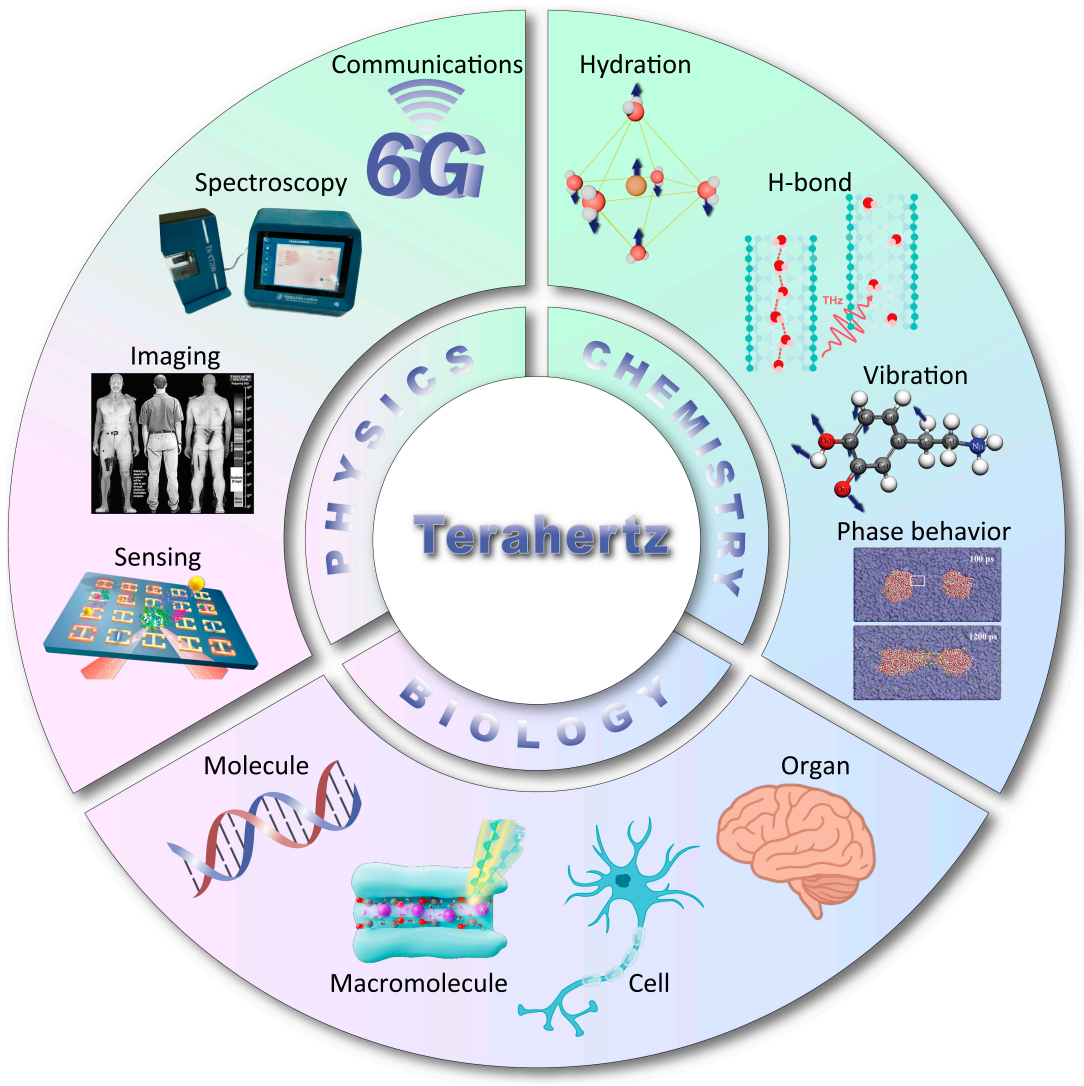
Schematic overview of THz science at the interface of physics, chemistry, and biology. THz radiation underpins a wide range of fundamental studies and applications, spanning spectroscopy, imaging, sensing, and wireless communications in physics; probing hydration dynamics, hydrogen bonding, molecular vibrations, and phase behavior in chemistry; and investigating biological systems from molecules and macromolecules to cells and organs.

Terahertz (THz) technology has rapidly emerged as a powerful and versatile tool in biophysics [1,2] and chemistry [3], offering a unique window into the molecular world by bridging the gap between fundamental scientific insights and cutting-edge technological applications [4], as shown in Fig. 1. THz wave, generally defined within the frequency range of 0.1 to 30 THz (corresponding to wavelengths from 3,000 to 10 μm), occupies the spectral region between microwaves and infrared light [5]. To better reflect the biophysical relevance of high-frequency electromagnetic interactions, the concept of generalized THz waves has been proposed, extending the range to 100 THz [6]. Once known as the “THz gap” due to historical challenges in generation and detection, this region has become increasingly accessible because of breakthroughs in ultrafast optics [7], photoconductive materials [8], quantum cascade lasers [9–13], and nonlinear optical crystals [14]. Importantly, the nonionizing nature of THz waves, their sensitivity to collective molecular motions, and their ability to penetrate various nonmetallic and nonpolar materials make them ideally suited for investigating biological and chemical systems at the mesoscale and molecular level. As a result, the interactions between THz radiation and biological systems have led to its expanding application prospects in fields such as water transport [15–17], medical diagnostics [18,19], neurobiology [20], and molecular dynamics (MD) [21].

The field of THz biophysics and chemistry provides valuable insights into how THz radiation interacts with molecules and reveals important aspects of biomolecular structure and function [22]. Many biologically relevant motions, such as protein conformational dynamics [23], molecular libration [24], hydrogen bonding vibration [25], hydration shell fluctuations [26,27], and torsion angle oscillations [28], occur in the THz frequency range. By probing these dynamics, THz techniques offer insights into biological processes that are otherwise difficult to observe. When the frequency of the external field matches that of these dynamic processes, THz radiation has the potential to influence both the structural and dynamic properties of molecules. Moreover, based on the specific responses of biomolecules to THz radiation, THz radiation holds promise for biomedical diagnostics and therapeutics [29]. Specifically, THz spectroscopy and imaging can detect subtle changes in tissue composition associated with early-stage cancers or neurodegenerative diseases [18,30], while emerging evidence suggests that THz radiation can modulate cellular and neuronal activity [31], opening avenues for noninvasive therapeutic strategies. The development of THz biophysics and chemistry also exemplifies the importance of interdisciplinary collaboration, requiring joint efforts across physics, chemistry, biology, and materials science. With a deeper understanding of the underlying mechanisms of THz-biological interactions, along with continuous breakthroughs in THz source development [12], signal interpretation [32], and system integration [33], this field is poised for further transformative progress. Despite the considerable progress achieved in this field, comprehensive reviews are still lacking.

**Fig. 2.**
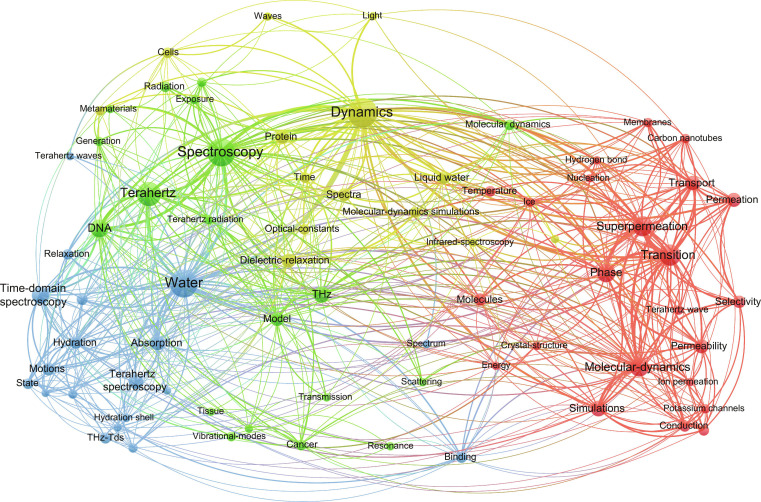
Keyword co-occurrence network related to THz biophysics and chemistry, visualized using VOSviewer. The network is constructed from bibliometric data of research articles published in recent years. Node size represents keyword frequency, while link thickness indicates co-occurrence strength. Colors represent distinct thematic clusters.

Herein, we aim to provide a comprehensive overview of the current landscape of THz biophysics and chemistry, with a scope that covers the generalized THz wave range. To systematically capture the current landscape of THz biophysics and chemistry, we conducted a bibliometric analysis of recent publications in this interdisciplinary field using VOSviewer v1.6.20. The resulting keyword co-occurrence network (Fig. 2) highlights major thematic clusters, including MD, water, ion conduction and channels, DNA, and THz spectroscopy, reflecting the central research directions and guiding the structure of this review.

## THz and Its Potential Relationship with Biology, Physics, and Chemistry

### Fundamental characteristics of THz wave

THz wave exhibits a unique set of characteristics that make it especially valuable for investigations in physics [13,34], biology [35], and chemistry [36,37]. As a nonionizing form of electromagnetic radiation, THz waves lack sufficient energy to break chemical bonds or ionize atoms, making them generally safe for biological systems under controlled exposure. A defining feature of THz radiation is its sensitivity to low-frequency molecular rotations and vibrations [38]. Its frequencies align with the natural vibrational and rotational modes of many molecules, particularly large biomolecules such as proteins, DNA, and water clusters, allowing THz wave to probe molecular structures and dynamics that are often inaccessible by other conventional techniques. Notably, THz radiation excels in detecting collective molecular motions—such as torsional oscillations, hydrogen-bond dynamics, and hydration shell fluctuations—that are intimately tied to biomolecular function [39,40]. Moreover, THz radiation can penetrate materials such as fabrics, plastics, and certain biological tissues while being strongly absorbed by water [41]. This selective penetration makes it potentially valuable for imaging hidden objects, detecting cancerous tissues, and security screening. Its strong interaction with polar molecules, especially water, both limits deep tissue penetration and provides a powerful means to study water-rich environments like hydrated proteins and living cells [42]. These unique attributes make THz wave an indispensable tool for exploring the dynamic and functional landscapes of biological and chemical systems, laying the groundwork for the mechanistic and application-oriented discussions that follow.

### THz radiation for investigating biomolecular structure–function relationships

Biomolecules such as proteins, nucleic acids, and lipids possess intricate structures whose functions are dictated by complex intra- and intermolecular interactions across diverse spatial and temporal scales [43]. A central principle in structural biology is that function arises from both molecular structure and its dynamic fluctuations. The dielectric response of matter in the THz range encodes rich information about molecular composition and organization, enabling label-free sensing and structural characterization [39]. THz wave is uniquely suited to probing low-frequency molecular motions related to structure, such as hinge bending and interdomain vibrations, which underlie essential biological processes like enzyme catalysis, ligand binding, and allosteric regulation. These collective vibrational modes, often involving noncovalent interactions such as hydrogen bonding and van der Waals forces, fall within the THz regime, enabling direct coupling between THz waves and biomolecular dynamics.

Similarly, nucleic acids and lipid assemblies exhibit THz-active modes, including backbone torsions, base-pair breathing, and hydration-dependent fluctuations in DNA or RNA [44,45], as well as collective headgroup and acyl chain motions in lipid bilayers. Such motions influence structural flexibility, interaction potentials, and functional responsiveness. Unlike traditional structural techniques (e.g., x-ray crystallography or cryo-electron microscopy) that capture static snapshots [46], THz spectroscopy provides dynamic, real-time insight into conformational ensembles and their transitions under near-physiological conditions. In this way, THz wave offers a powerful, noninvasive window into the functional dynamics of biomolecules, complementing and extending our understanding of structure–function relationships in complex biological systems. Its ability to probe soft vibrational modes enables the detection of functionally relevant motions that are critical for biological activity, making it a powerful tool in the study of complex biomolecular systems.

### Molecular motions in the THz regime

**Fig. 3.**
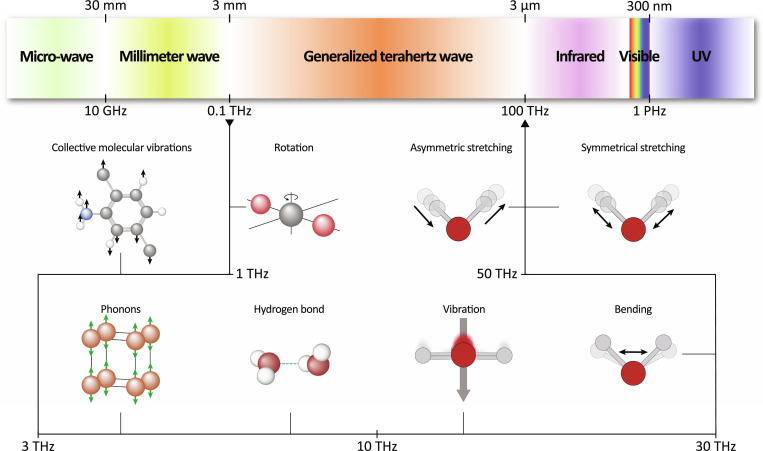
Representative electromagnetic spectrum and molecular motion couples to THz radiation. The highlighted generalized THz regime can reflect various inherent molecular motions, including collective molecular vibrations, phonons, rotational modes, hydrogen-bond dynamics, low-frequency bending, and stretching vibrations.

Figure 3 shows the electromagnetic spectrum and molecular motion couples to generalized THz wave. Molecular motions in the frequency range below 30 THz encompass low-frequency, collective vibrational and rotational phenomena that are fundamental to biological and chemical function [47]. This feature makes THz wave to couple to large-scale, delocalized dynamics such as torsional oscillations [43,44], hydrogen-bond network fluctuations [48], and inter- or intramolecular vibrations [49]. These motions often involve multiple atoms or entire molecular domains and are inherently anharmonic and thermally accessible at physiological temperatures, making them particularly relevant to biological systems. Molecular motions in the frequency from 30 to 100 THz primarily target localized bond vibrations, including bending and stretching [35,50]. In proteins, for example, normal mode analysis and MD simulations have identified THz-active modes such as hinge bending and global breathing motions closely tied to functional conformational shifts. Similarly, in nucleic acids, THz vibrations are associated with base-pair shearing, helical twisting, and backbone fluctuations, which are mechanisms integral to processes like DNA replication and molecular recognition. Understanding and harnessing these motions provide key insights into the fundamental physics of biological activity and offer promising opportunities in THz-driven applications, including molecular engineering, catalysis, and targeted therapeutics.

## THz–Biomolecule Interactions at the Molecular Level

### Fundamental principles of THz interaction with matter

The fundamental principles governing the interaction between THz radiation and matter provide critical insight into its unique capabilities for probing and manipulating molecular systems relevant to biophysics and chemistry. These interactions are fundamentally dictated by Maxwell’s equations and are shaped by the dielectric, conductive, and vibrational properties of the material. Unlike higher-frequency radiation that primarily couples to electronic transitions, THz radiation resonate with low-energy excitations such as rotational, torsional, vibrational, and collective modes [51], which are dynamical processes that are often central to molecular recognition, conformational changes, and biochemical function. These modes, typically lying within the THz frequency range, involve dipole moment fluctuations, making them particularly responsive to THz radiation. This property uniquely positions THz wave as a powerful tool to investigate soft matter systems, ranging from individual biomolecules to complex biological assemblies such as hydrated proteins, lipid bilayers, and tissues. The key physical parameter describing the THz response of a material is its complex permittivity, ε(ω) = ε′(ω) − iε″(ω), which encodes both the energy storage (real part) and dissipation (imaginary part) characteristics of the system [52]. In biological environments, pronounced frequency-dependent dielectric dispersion and absorption arise due to dipolar relaxation and vibrational resonances, often modeled by Debye or Lorentzian functions to relate spectral features to molecular motion.

At the molecular level, the interaction between THz radiation and matter is primarily mediated through the dynamic response of molecular dipole moments [43]. Resonant coupling occurs when the frequency of the incident THz radiation matches that of intrinsic vibrational or torsional modes of the molecule [53]. This resonance enables efficient energy transfer from the external field to internal molecular motion, selectively enhancing specific vibrational or rotational degrees of freedom [25]. Molecules possessing permanent dipole moments undergo instantaneous changes during conformational fluctuations, such as stretching or torsion, that result in characteristic absorption features in the THz and infrared regions. Even in the absence of external excitation, thermal fluctuations cause dipole moment variations [54], but when resonant conditions are met, the THz radiation imposes a time-varying torque on the dipole [55], driving energy accumulation and dynamic reconfiguration. These mechanisms underpin the following analysis of biomolecular systems, from water to complex macromolecules.

### THz and water

Water, as a universal solvent and vital component of biological systems, has been the focus of extensive research regarding its interaction with THz radiation. Based on the assumption of an ordered arrangement of water molecules on the neuronal surface, theoretical studies have firstly predicted intrinsic vibration modes of electromagnetic radiation on the surface located in the THz and far-infrared range, which may contribute to neural signal generation and transmission [6]. These findings have sparked interest in the relationship between THz radiation and water.

**Fig. 4.**
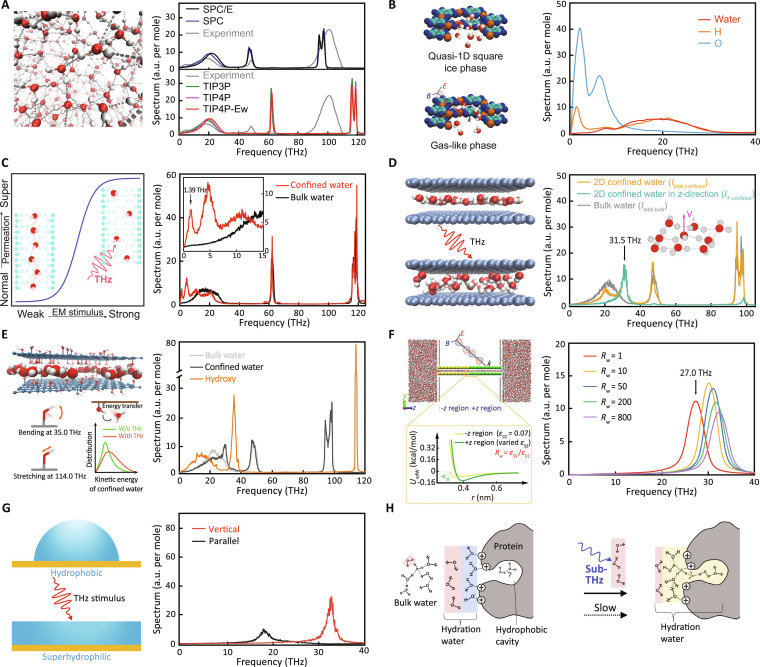
Interaction between THz radiation and water. (A) Relationship between the hydrogen-bond network and the THz absorption spectrum [56]. (B to G) Frequency-specific THz radiation modulates the structure and dynamics of water confined within MOF pores [57] (B), CNTs [58] (C), graphene slits [17] (D), GO membranes [59] (E), heterogeneous-wettability nanochannels [16] (F), and at interface [67] (G). (H) Effect of sub-THz irradiation on the hydration of heterogeneous protein surfaces [23].

MD simulations have provided valuable insights into this relationship. One example involves the strong coupling between THz signals and hydrogen bonding. Duan et al. [56] indicated that the peak positions of water absorption spectra in the THz range are directly correlated with hydrogen-bond lifetimes (Fig. 4A). Another important area of investigation involves confined water systems, such as those found in carbon nanotubes (CNTs) or graphene-based membranes. Water in these environments exhibits distinct THz absorption characteristics compared to bulk water, leading to remarkable responses under THz radiation. Studies have proposed frequency-specific effects on water transport. Zhu et al. [17,57,58] predicted THz-induced phase transitions in confined water within metal–organic framework (MOF) (Fig. 4B), CNTs (Fig. 4C), and graphene slits (Fig. 4D), resulting in substantial enhancements in water flow by disrupting hydrogen-bond networks. Sun et al. [59,60] suggested up to a 141-fold enhancement and a 5-fold increase in water permeability across graphene oxide (GO) membranes under THz radiation. These enhancements were attributed to THz-induced resonance with internal hydroxyl and edge-functionalized groups on the GO membranes, which disrupted the hydrogen-bond network (Fig. 4E). Yang et al. [61] indicated enhanced absorption due to molecular alignment in confined water. Research by Zhang et al. [62,63] and Zhao et al. [64] predicted that THz radiation could trigger ultrafast conductivity and flow transitions through resonant coupling (Fig. 4F). More recently, Wu et al. [65,66] suggested that THz radiation could melt structured water phases in nanotubes and promote single-file water transport in narrow channels, offering promising strategies for future nanofluidic device design. Similarly, Zhu et al. [67] predicted that frequency-specific THz radiation might restructure interfacial water, inducing a wetting transition from hydrophobic to hydrophilic (Fig. 4G). Additionally, computational studies on THz radiation–water interactions have broadened these insights to applications in energy and environmental technologies, such as gas hydrate dissociation and desalination. Zhu et al. [68] and Fang et al. [69] indicated that specific THz frequencies could disrupt hydrogen bonding in methane hydrates, improving their decomposition and solubility. In the context of desalination, Zhu et al. [57] and Wu et al. [70] suggested improved water flux in CNT- and MOF-based membranes under THz radiation while maintaining effective ion rejection.

Experimental studies have provided crucial validation and complementary insights to theoretical predictions regarding THz–water interactions. In the context of hydration dynamics and hydrogen bonding, Heyden et al. [41] identified specific THz absorption peaks linked to solvation shell dynamics, highlighting the sensitivity of THz signal to molecular-level water structure. Other studies demonstrated that THz radiation and protein environments modulate water polarization and hydrogen bonding, influenced by factors like ionic viscosity (Fig. 4H) [23,71]. Zhang et al. [72] employed 2-dimensional (2D) THz rotational spectroscopy to detect transient water complexes, while Elgabarty et al. [25] used pump-probe methods to follow energy transfer within hydrogen-bond networks. Cross-disciplinary approaches have also proven valuable. Tokunaga et al. [73] integrated THz and nuclear magnetic resonance techniques to examine hydration dynamics, and Shiraga et al. [74] combined THz spectroscopy with dielectric relaxation to gain broader insights into water behavior at different scales. In confined water systems, Penkov et al. [26] used THz time-domain spectroscopy (THz-TDS) to examine hydration in lipid membranes, revealing temperature-dependent changes in free water and stronger intermolecular interactions during phase transitions. Regarding dissociation and phase transitions, Bi et al. [75] demonstrated that 1-THz pulses can destabilize water–oil interfaces for demulsification, while Ma et al. [76] showed that THz radiation can control water phase transitions in nanochannels, enabling precise regulation of freezing and melting.

### THz and biological ion channels

Ion channels are essential for numerous biological processes, including nerve signaling, muscle contraction, and cellular communication. A groundbreaking discovery has been made on modulating the ion channel of TRPV1 (transient receptor potential vanilloid 1) for pain relief via solvent molecules, which potentially provides alternative strategies for pain management [77]. Moreover, emerging research suggests that THz radiation can interact with these channels and influence their behavior. Understanding these effects is important for evaluating potential biological impacts and exploring future biomedical applications. Recent reviews, such as that by Liu et al. [78], have summarized progress in THz modulation of neuronal ion channels, highlighting both functional changes and potential health implications.

Before reviewing specific advances on the effects of THz radiation on ion channel, it is important to provide contextual understanding. In reality, the strong absorption of THz radiation by water can substantially influence its interaction with ion channels. However, some of these advances are based on simulations, where MD simulations are typically performed at the nanometer to submicrometer scale and often neglect the attenuation of THz radiation reaching ion channels. Therefore, in practical applications, careful optimization of the THz frequency is essential to avoid regions of strong water absorption, thereby maximizing the propagation of the radiation. For instance, Liu et al. [79] demonstrated that, with appropriately selected frequencies, THz radiation can affect ion channel activity located more than 300 μm away.

**Fig. 5.**
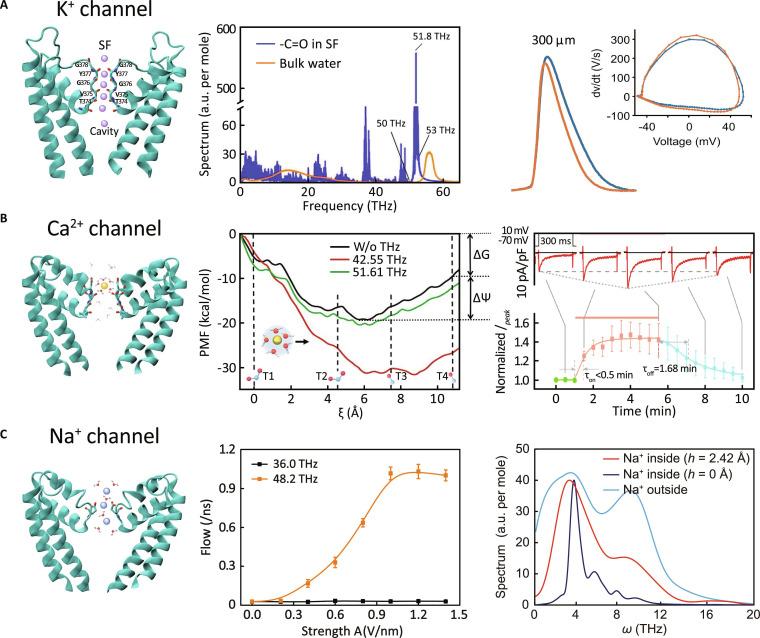
Interaction between THz radiation and ion channels. (A) Potassium channel: Illustration highlighting carbonyl groups in the selectivity filter, based on the Protein Data Bank (PDB) structure 1K4C, comparison of THz absorption between these carbonyls and bulk water (middle, [86]), and modulation of K^+^ currents by frequency-specific THz radiation (right, [79]). (B) Calcium channel: Illustration highlighting carboxyl and carbonyl groups in the selectivity filter, based on the PDB structure 4MVQ; modulation of Ca^2+^ currents by frequency-specific THz radiation shown in simulations (middle, [89]) and experiments (right, [92]). (C) Sodium channel: Illustration highlighting carboxyl groups in the selectivity filter, based on the PDB structure 3RVY; modulation of Na^+^ currents by frequency-specific THz radiation (middle, [93]) and comparison of Na^+^ spectra inside versus outside the channel (right, [95]).

A growing number of studies have examined the influence of THz radiation on potassium (K^+^) channels (Fig. 5A). MD simulations suggest that THz radiation at specific frequencies could modulate the vibrational dynamics of channel residues and thereby affect ion transport properties. For instance, Zhao et al. [80] predicted that 51.8-THz radiation could excite carbonyl group vibrations in the selectivity filter of the K_V_1.2 channel, potentially enhancing potassium flow and improving the channel’s stability. Wang et al. [81] suggested that the direction of the THz radiation could affect K^+^ transport in the KcsA channel, with ion flux varying based on the angle of radiation, a finding that could inform targeted cancer therapies. Wu et al. [79,82] identified 53.60 THz as an optimal frequency for boosting potassium permeability by calculating 2D infrared spectroscopy. This frequency nearly doubled ion flow and increased selectivity 10-fold due to resonant coupling with carbonyl vibrations. Other modeling efforts by Hu et al. [83] indicated that THz radiation could increase ion current 4-fold compared to control conditions. Wang et al. [84] further suggested that ion coherence under THz excitation might enhance ion transport efficiency with reduced energy dissipation. Theoretical investigations incorporating quantum effects such as tunneling and zero-point energy might explain near-diffusion-limited transport under THz influence [85]. Additionally, Wang et al. [86] suggested that 51.87-THz radiation could stabilize interactions within the selectivity filter via Y78 residue vibrations, thereby enhancing potassium flow. Sun et al. [87] predicted that 53.7-THz radiation might modify the secondary structure of KcsA channels, boosting K^+^ transport. Meanwhile, Ding et al. [88] proposed that 15-THz radiation could disrupt hydrogen bonds and suppress the “soft knock-on” mode in K_V_1.2, increasing ion flux by 68%.

Research on calcium (Ca^2+^) channels also shows promising results, as shown in Fig. 5B. Simulation results provide valuable predictions regarding the modulation of calcium transport by THz radiation. For instance, Li et al. [89] originally predicted that resonant THz excitation dramatically improved calcium selectivity and conductance by almost 5-fold by strengthening hydrogen bonds and lowering transport energy barriers, with promising therapeutic applications in calcium-related disorders and cancer. Similarly, Guo et al. [90] indicated that THz radiation could accelerate Ca^2+^ movement by interacting with naturally emitted ionic radiation. Moreover, Sun et al. [91] proposed that the applied THz frequency coincides with the symmetric vibrational mode of the carboxyl group within the selectivity filter, which reduces the potential of mean force of Ca_V_2.1 and facilitates Ca^2+^ transport through the channel. Validating these computational findings, experimental work in 2024 demonstrated that 42.5-THz stimulation enhanced Ca_V_1.2-mediated calcium influx and activated CREB/c-Fos signaling pathways, without requiring genetic modifications [92].

Sodium (Na^+^) channels have also gained attention due to their potential responsiveness to THz radiation (Fig. 5C). Computational studies suggest that specific-frequency THz radiation may enhance sodium permeability through unique energy transfer mechanisms. For instance, Zhao et al. [93] performed simulations on an artificial model, indicating that THz radiation at frequency of 48.2 THz could increase sodium permeability by over 30-fold. Similarly, Song et al. [94] predicted several new characteristic THz frequencies that enhance the ion permeability of Na_V_1.5 and K_V_1.2, likely influenced by the intrinsic oscillatory motions of permeating ions in the selectivity filter or by specific chemical groups within the channels. In addition, Zhu et al. [95] developed synthetic sodium channels inspired by natural designs, suggesting that frequency-synchronized vibrations inside and outside the channels may improve ion flow within the THz band.

### THz and DNA, and proteins

**Fig. 6.**
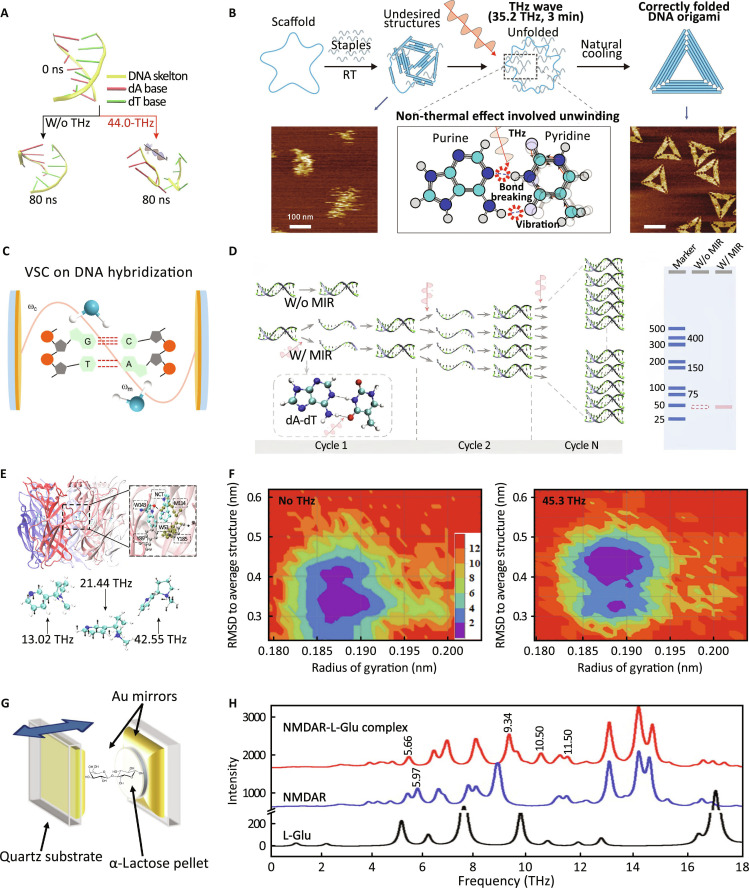
Interaction between THz radiation and DNA/biological molecules. (A) Frequency- and intensity-specific THz radiation accelerates DNA unwinding [96]. (B) Experimental validation of duplex unwinding promotion in a DNA origami assembly system [97]. (C) VSC between DNA molecules and optical cavity photons [99]. (D) THz photons markedly enhance PCR amplification efficiency [100]. (E) Specific THz radiation significantly destabilizes protein–ligand interactions [103]. (F) A 45.3-THz radiation reduces the binding free energy between choline and choline acetyltransferase [104]. (G) Strong coupling between THz cavity modes and vibrational modes in α-lactose [47]. (H) NMDAR–ligand systems exhibit high structural sensitivity to THz perturbations [106].

DNA and proteins are the fundamental macromolecules of life. The interaction between THz radiation and DNA has attracted significant research interest due to its ability to modulate molecular stability, induce unwinding, and influence hybridization behavior. A breakthrough theoretical study by Chang’s group [96] predicted that a 44.0-THz electric field could resonantly break hydrogen bonds in purine-rich DNA sequences, remarkably accelerating duplex unwinding by more than 20-fold through nonthermal mechanisms (Fig. 6A). Two years later, the same group validated this prediction experimentally by showing that THz irradiation at specific frequencies can effectively promote duplex unwinding in a DNA origami assembly system, thereby enhancing nanostructure yield (Fig. 6B) [97]. These pioneering efforts have inspired subsequent investigations into DNA–THz interactions.

Theoretical calculations have revealed several novel mechanisms. For instance, it has been proposed that THz radiation could control proton transfer within DNA base pairs, suggesting potential applications in modulating catalytic reactions at the molecular scale [98]. Beyond DNA duplexes, RNA structures have likewise been shown to respond to THz irradiation. THz radiation has been reported to influence the unfolding dynamics of RNA hairpins under mechanical extension [44]. Building upon these conceptual frameworks, subsequent experiments have achieved breakthroughs. It has demonstrated that vibrational strong coupling (VSC) between DNA molecules and optical cavity photons can reduce the thermodynamic stability of DNA duplexes, with this effect further modulated by Mg^2+^ ion concentration (Fig. 6C) [99]. In a complementary experimental approach, quantum vibrational coupling with THz photons has been shown to significantly enhance the efficiency of polymerase chain reaction (PCR) amplification, with the degree of enhancement dependent on the length of the double-stranded DNA (Fig. 6D) [100]. Moreover, studies of double-stranded DNA adsorbed on gold [Au(111)] surfaces revealed THz-induced perturbations in hydrogen bonding and changes in vibrational spectral characteristics [101]. Additionally, earlier experimental work provided biological validation of low-frequency vibrational modes in nucleic acids, identifying multiple well-resolved phonon resonances in short DNA duplexes using THz Fourier transform infrared spectroscopy [102].

Extending beyond DNA, THz radiation has demonstrated a remarkable ability to modulate protein conformations and ligand-binding dynamics through nonthermal mechanisms by altering hydrogen bonding, dipolar orientations, and collective vibrational modes. MD simulations have been employed to provide mechanistic insights into protein–ligand interactions under THz irradiation. Notably, specific THz frequencies have been shown to significantly modulate these interactions. For instance, it has been reported that specific THz radiation (e.g., 13.02 THz) might significantly destabilize protein–ligand interactions, markedly increasing ligand dissociation probabilities once a critical field threshold is exceeded (Fig. 6E) [103]. Similarly, it was predicted that 45.3-THz radiation could reduce the binding free energy between choline and key catalytic residues in choline acetyltransferase, potentially impairing enzymatic synthesis of acetylcholine (Fig. 6F) [104].

Experimental studies provide strong support for these computational findings on THz-induced modulation of protein conformations and ligand-binding dynamics. For example, electron paramagnetic resonance (EPR) measurements have revealed that THz irradiation alters nitric oxide affinity and paramagnetic site mobility in serum albumin, reflecting conformational changes in protein functional groups [105]. Another study has shown that strong coupling between THz cavity modes and vibrational modes in α-lactose resulted in 68-GHz Rabi splitting, suggesting the feasibility of polaritonic control over protein dynamics (Fig. 6G) [47]. Functional protein complexes such as the *N*-methyl-d-aspartate receptor (NMDAR)–ligand system have also shown high structural sensitivity to THz perturbations; THz-TDS and quantum simulations revealed that water-mediated low-frequency vibrations play a key role in regulating receptor stability (Fig. 6H) [106]. Moreover, THz spectroscopy has been applied to detect structural alterations in kidney proteins post-x-ray exposure, demonstrating its sensitivity to changes in hydrogen bonding and secondary structure content [107]. Beyond static structure, protein folding dynamics have been probed by kinetic THz absorption (KITA) spectroscopy, capturing submicrosecond hydration-coupled transitions during early folding events [108]. Additionally, environmental factors such as hydrogen-rich water have been shown to modulate enzyme structure and activity via localized hydrophobic interface remodeling, as evidenced by altered THz signatures in pepsin [109]. This arises because molecular vibrations in the THz regime correspond to the timescale of water reorientation and hydrogen-bond rearrangements, whereby hydrogen molecules induce collective domain motions and structural changes in the enzyme. Similarly, THz radiation has also been found to influence both the activity and conformation of alkaline phosphatase nonthermally [110]. Importantly, in the context of pathological aggregation, high-frequency THz radiation (e.g., 34.88 THz) has been experimentally shown to delay amyloid-β (Aβ) fibril formation by approximately 80% without causing cytotoxicity in normal cells [111], and further computational work indicates that 42.55-THz stimulation enhances β-sheet formation in Aβ_42_ monomers and dimers while mildly stabilizing protofibrillar assemblies, offering molecular-level insight into the potential of THz radiation as a modulator of neurodegenerative processes [112]. Interestingly, Schroer et al. [113] found no large-scale structural changes in proteins under THz irradiation, which suggests that THz effects may need to match frequencies and sufficient strength, and any observable changes could be transient, local, or outside the studied spectral and power range. In other words, radiation intensity plays a role comparable to frequency resonance in determining molecular responses. The intensity determines the amount of energy delivered to the molecules. At low intensities, the energy absorbed by the molecules may be insufficient to disrupt the weak interactions that maintain the stability of the molecular system, and thus will not significantly affect molecular structure or dynamics [16,17,43]. This further highlights the importance of carefully controlling both the frequency and intensity of THz radiation in practical applications.

## THz Biological Effects

### THz and cell-level effects

**Fig. 7.**
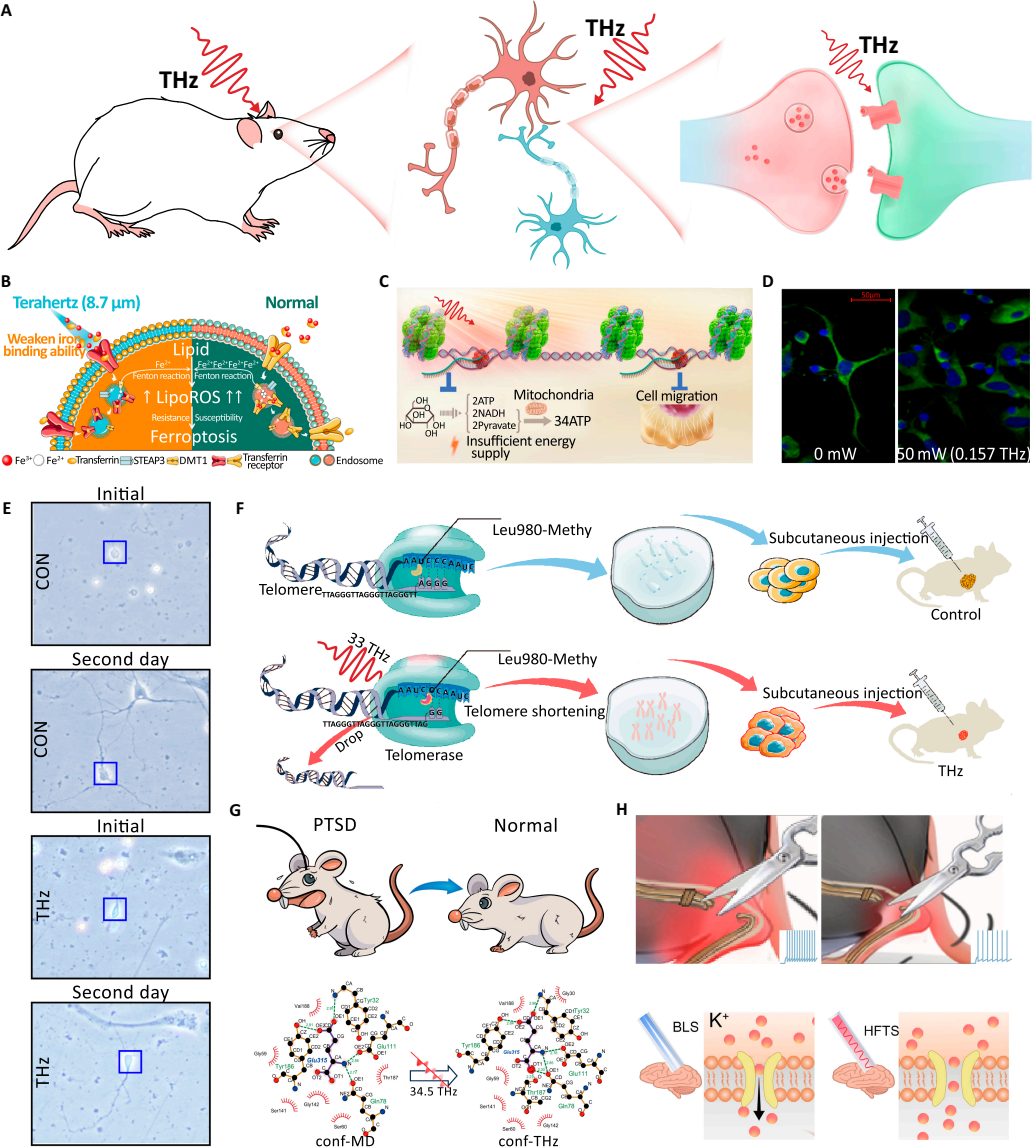
THz biological effect. (A) THz photons improve cognitive functions [150]. (B) THz wave-mediated inhibition of ferroptosis [114]. (C) Suppression effects of THz radiation on cancer cell [115]. (D) Expression of PSD-95 protein in neurons after exposure to THz radiation [128]. (E) THz radiation promotes neuronal outgrowth [134]. (F) THz photons inhibit telomerase activity [30]. (G) THz photons improve cognitive functions [150]. (H) THz stimulation alleviates neuropathic pain [142].

Accumulating evidence has demonstrated that THz radiation elicits a range of biological effects at the cellular level (Fig. 7A). These effects encompass alterations in cell viability, membrane integrity, ion transport, neural excitability, gene expression, and intracellular signaling, largely through nonthermal mechanisms. On the one hand, recent studies have highlighted that THz radiation can regulate cell viability and cell death pathways. For example, Li et al. [114] demonstrated through MD simulations and chromogenic assays that THz radiation inhibits ferroptosis by disrupting ferric ion binding to transferrin, effectively reducing ferroptosis-related markers in both cell and animal models (Fig. 7B). In another study, Chang’s group [115] demonstrated firstly that high-frequency THz radiation inhibited cancer cell migration and glycolysis through epigenetic modifications, with great therapeutic potential (Fig. 7C). Moreover, Yin et al. [30] discovered that specific-frequency THz photon irradiation significantly suppressed telomerase activity, effectively reduced the viability of 4T1 and MCF-7 cancer cells, and amazingly diminished tumorigenicity in vivo. Chen et al. [116] further reinforced this finding by identifying 1131 differentially expressed genes in THz-exposed leukemia K562 cells, enriched in proliferation and apoptosis pathways such as mitogen-activated protein kinase (MAPK) and Notch signaling. Niu et al. [117] observed that 53.5-THz radiation not only enhanced cell proliferation but also broadly altered gene expression profiles, highlighting the multifaceted nature of THz-induced bioactivity.

On the other hand, THz radiation also exerts direct influence on cell membrane structure and ion transport. Bo et al. [118] analyzed the energetic behavior of Na^+^, K^+^-ATPase (adenosine triphosphatase) under THz unipolar stimulation, finding reduced ionic flow and minimal energy consumption, which suggests efficient ion pump modulation. Complementary MD simulations by Tang et al. [119] and Vernier et al. [120] demonstrated that THz electric fields can induce electroporation in phospholipid membranes, with the frequency and waveform of the field strongly influencing pore formation via hydrogen-bond resonance. Experimental evidence from Lin et al. [121] supports these findings, as 3.1-THz irradiation was found to enhance membrane fluidity and phagocytic function in macrophages, corroborated by THz-TDS and density functional theory simulations. Furthermore, Tan et al. [122] provided mechanistic insights by demonstrating that THz photons at specific frequencies restore action potential firing in neurons under adenosine triphosphate (ATP) depletion, suggesting a photon-assisted energy transfer mechanism related to ATP phosphate bond cleavage.

Moreover, neural and stem cells also appear particularly responsive to THz irradiation. Wang et al. [123] performed flow cytometry and immunofluorescence analysis on human and mouse neural stem cells (NSCs) and revealed dose-dependent changes in proliferation, apoptosis, and DNA damage, with human neural stem cells (hNSCs) being more sensitive to THz radiation. In a related study, Zhao et al. [124] investigated rat hippocampal neurons and found that both 0.12- and 0.157-THz irradiation altered their morphology and function (Fig. 7D). Additionally, Tang et al. [125] showed that nicotine modulates the THz absorption characteristics of neural cells in a dose-dependent manner, linking these changes to morphological and proliferative alterations in HEB and U87 cells. Meanwhile, Zhang et al. [126] used atomistic MD simulations to investigate dielectric dispersion across lipid bilayers and found that the 55- to 85-THz range supports advantageous wave propagation due to subnanometer dielectric heterogeneity.

Additionally, cellular transcriptional and microbial responses to THz radiation further deepen our understanding of its biological effects. Peltek et al. [127] revealed that while heat shock protein genes remained unchanged, THz radiation modulated genes related to transition metal ion homeostasis in *Geobacillus icigianus*, suggesting specific gene expression sensitivity. Nonthermal THz effects on microbial viability were reported by Boev et al. [128], who demonstrated cell death in microbial suspensions via pulse-THz radiation using multimodal analyses. Likewise, Kakikawa et al. [129] showed that 0.46-THz radiation significantly enhanced bleomycin cytotoxicity in *Escherichia coli* without increasing membrane permeability, implying intracellular modulation of drug activity. Supporting this, Grognot and Gallot [130] used THz attenuated total reflection imaging to track real-time protein leakage in saponin-permeabilized live epithelial cells, confirming the utility of THz radiation in studying dynamic membrane processes.

### THz and neurobiology

The impact of THz radiation on neurobiological processes has also been explored. Studies have shown that THz waves can modulate the activity of neurons and synapses, potentially influencing neural signaling and positioning it as an emerging technique for precision neuromodulation [131]. Accumulating evidence indicates that THz waves, especially in the range of 0.1 to 50 THz, can elicit diverse biological responses in neural tissues, both in vitro and in vivo. Liu et al. [132] provided a comprehensive review of THz neurobiological studies, covering in vivo and in vitro neuronal responses, THz-based neuromodulation via microelectrode arrays, and computational models of neural signal encoding and decoding. Experimental studies have shown that specific THz frequencies can promote neuronal growth, enhance synaptic transmission, and modulate signal firing patterns. For instance, Zhong et al. [133] demonstrate that stimulation with 34.5-THz photons promotes neuronal growth and synaptogenesis by up-regulating the cyclic adenosine monophosphate signaling pathway and adenylyl cyclase type 1 activity. Ma et al. [134] demonstrated that 0.138-THz radiation significantly enhances synaptic transmission in the hippocampal CA1 region and promotes neuronal outgrowth, possibly through cumulative exposure effects (Fig. 7E). Similarly, Tan et al. [122] reported that certain THz photons can restore the action potential frequency reduced by ATP deficiency, revealing an alternative energy-supply pathway in neurons. These findings suggest that THz radiation may serve as a novel neuromodulatory modality, with both therapeutic and diagnostic potential.

Complementing experimental observations, theoretical and computational investigations have elucidated the underlying mechanisms of THz–neuron interactions, particularly in signal propagation, waveguide behavior, and resonance phenomena. Zhang et al. [135] developed a loss-amplification model for myelinated neurons and showed that THz signals (55 to 75 THz) could maintain intensity through loss-compensated propagation, which is disrupted by demyelination. Liu et al. [136,137] revealed through infrared microspectroscopy that the myelin sheath functions as an infrared dielectric waveguide with energy amplification at the nodes of Ranvier, and later extended this concept using a quasi-quantum computational model to describe high-frequency electromagnetic propagation in myelinated fibers. Zeng et al. [138] further established a multilayer electromagnetic simulation demonstrating that myelinated axons behave as low-loss, low-dispersion waveguides with wavelength dependence on axon geometry. These findings, while currently based on theoretical models, provide valuable insights and offer a promising basis for future experimental directions. More recently, Guo et al. [139] proposed a weak resonance effect of THz waves in nerve cells, depending on cell size and dielectric properties, using THz-TDS and finite-difference time-domain (FDTD) modeling. Computational approaches such as MD simulation and density functional theory (DFT) have also been employed to investigate neurotransmitter aggregation and choline binding modulation in enzymatic pathways, further highlighting the molecular-scale specificity of THz interactions [104,140]. These multi-scale theoretical models contribute to a deeper understanding of how THz radiation may influence signal fidelity, neurotransmitter behavior, and cellular communication in the nervous system.

In parallel with mechanistic studies, THz-based technologies are being explored for both neuromodulation and neurodiagnostics. Tan et al. [141] showed that 30- to 45-THz photons can resonate with neurotransmitters, enhance synaptic currents, and alter neuronal activity through in vivo imaging and quantum chemistry simulations. Peng et al. [142] demonstrated that 36-THz stimulation alleviates neuropathic pain via K_V_1.2-mediated potassium conductance, while Chang’s group [143] revealed profoundly that 34-THz waves remarkably relieve glutamatergic neuron hyperactivity in comorbid pain and anxiety by modulating hydrogen bonds in glutamate–GluA2 interactions. Although these findings are based on preclinical mouse experiments and computational analyses, they provide promising insights into THz-mediated pain management, motivating further experimental validation. Other studies such as Sun et al. [144] identified CaMKIIδ as a key mediator of THz-enhanced synaptic plasticity via nuclear factor κB (NF-κB) signaling, while Liu et al. [79] and Zhao et al. [145] further highlighted nonthermal, reversible, and frequency-specific effects of THz on neurons and glia. Meanwhile, diagnostic applications are gaining traction, as Chernomyrdin et al. [146] reviewed the prospects of THz imaging in detecting neurodegenerative diseases, tumors, and trauma, and Wang et al. [147] developed a THz chemical microscope for label-free dopamine sensing using aptamer recognition. Notably, Zhu et al. [148] introduced a THz-triggered dedocking method showing that 44.5-THz waves can dissociate dopamine from receptors by disrupting weak hydrogen bonds, suggesting a potential intervention strategy for neurodegenerative metabolite aggregation. Collectively, these advances illustrate the transformative potential of THz radiation in neuromodulation, neurochemical sensing, and brain disease therapy, although further studies are required to address safety, selectivity, and clinical translation.

### THz and therapy

The therapeutic potential of THz radiation has been investigated in various contexts, with studies demonstrating its ability to modulate biological processes at the molecular, cellular, and neural levels. Among these, cancer therapy represents a particularly promising application. For example, Yin et al. [30] achieved a breakthrough by showing that 33-THz irradiation significantly inhibited telomerase activity in 4T1 and MCF-7 breast cancer cells by up to 80%, resulting in cellular aging, apoptosis, DNA damage, and a remarkably 70% reduction in tumorigenicity in mice (Fig. 7F). Beyond direct anti-tumor effects, Cheon et al. [149] explored the use of THz spectroscopy for clinical cancer imaging, emphasizing its nonionizing nature, water sensitivity, and the challenges of attenuation and scattering, which could be mitigated using techniques such as cryogenic treatment and nanoparticle enhancement. Collectively, these studies indicate that THz radiation holds promise not only as a therapeutic agent but also as a diagnostic modality in oncological contexts.

In addition to cancer, THz and mid-infrared radiation have shown promise in treating neurological and psychiatric disorders. Chang’s group [150] applied THz photons to the hippocampal CA3 subregion to excitingly alleviate the posttraumatic stress disorder (PTSD) symptoms extremely difficult to treat and to restore cognitive function in rats by effectively enhancing protein expression and receptor–ligand binding, all through nonthermal mechanisms (Fig. 7G). Similarly, Peng et al. [142] demonstrated that 36-THz stimulation suppressed excitability of anterior cingulate cortex pyramidal neurons in a neuropathic pain model, through enhanced potassium conductance, highlighting a new optical neuromodulation strategy for pain relief (Fig. 7H). In psychiatric pharmacotherapy, Liu et al. [151] introduced a mid-infrared photonic therapy targeting KCNQ2 potassium channels in the auditory cortex to normalize hyperexcitability in tinnitus, providing evidence through molecular simulations and electrophysiological recordings. Complementary findings by Qi et al. [152] revealed that THz radiation improved behavioral outcomes related to anxiety, depression, and sociability in mice. Furthermore, Li et al. [153] proposed that 4.0-THz radiation could accelerate the dissociation of high-affinity antipsychotic drugs from their receptors, potentially reducing side effects associated with prolonged receptor binding. These converging lines of evidence position THz and adjacent frequency radiation as powerful, frequency-specific tools for modulating neural circuitry and biochemical targets, with significant therapeutic implications for mental and neurological disorders.

## Experimental Methods for Detection and Characterization in THz Biophysics and Chemistry

### Spectroscopy

THz spectroscopy has emerged as a powerful, label-free technique for the detection and characterization of biomolecular structures, interactions, and dynamics. The unique sensitivity of THz radiation to low-frequency collective vibrational modes enables the probing of hydrogen bonding networks, hydration dynamics, and conformational transitions in complex biological systems. The advancement of this field can be understood by examining the evolution of its fundamental insights, technological platforms, and diverse applications, which are comparatively summarized across these 3 thematic dimensions in Table 1.

**Table 1.** Thematic summary of advances in spectroscopy

Research focus	Author and year	Method	Sample	Main finding
Hydration and protein dynamics	Keutsch and Saykally [155]	THz spectroscopy	Water clusters	Probed cooperative H-bonding
	Heugen et al. [154]	THz spectroscopy	Hydration shells	Detected retarded H-bond dynamics
	Penkov [156,157]	THz-TDS	Protein hydration layers	Linked hydration dielectrics to function
	Nibali et al. [159]	MD simulations	Proteins	Identified collective protein vibrations
Advanced platforms	Soussi and Chalopin [160]	THz spectroscopy	Lipid bilayers	Distinguished lipids via dipole orientation
	Tang et al. [161]	Microfluidic THz system	Huntingtin gene	Detected gene mutations
	Yang et al. [163]	Microfluidic THz system	Protein solutions	Analyzed proteins in solution
	Tang et al. [162]	Droplet THz microfluidics	Coagulation factor VIII	Uncovered calcium-dependent domain dynamics
	Elayan et al. [164]	Theoretical modeling	THz nanoantennae	Proposed conformational control via nanoantenna
	Wu et al. [106]	Air plasma THz-TDS + DFT/ONIOM	l-Glu and NMDAR	Revealed solvent-mediated vibrational coupling
Biomolecular quantification and fingerprinting	Chen et al. [165]	THz spectroscopy	Tartaric acid enantiomers	Discerned chiral isomers
	Morales-Hernández et al. [27]	THz-calorimetric correlation	Carbohydrates	Quantified carbohydrate hydration
	Zhu et al. [166]	THz spectroscopy + docking	Dopamine	Linked dopamine conformation to spectra
	Ding et al. [167]	THz spectroscopy	Colloids	Probed colloidal behavior.
	Hou et al. [168]	THz-TDS	l-Arginine (aqueous)	Detected aqueous arginine
	Wang et al. [169]	THz-TDS	l-Arginine (solid, hydrated)	Correlated spectra with hydration state
	Xue et al. [170]	THz-TDS	Human urine	Diagnosed proteinuria

**Fig. 8.**
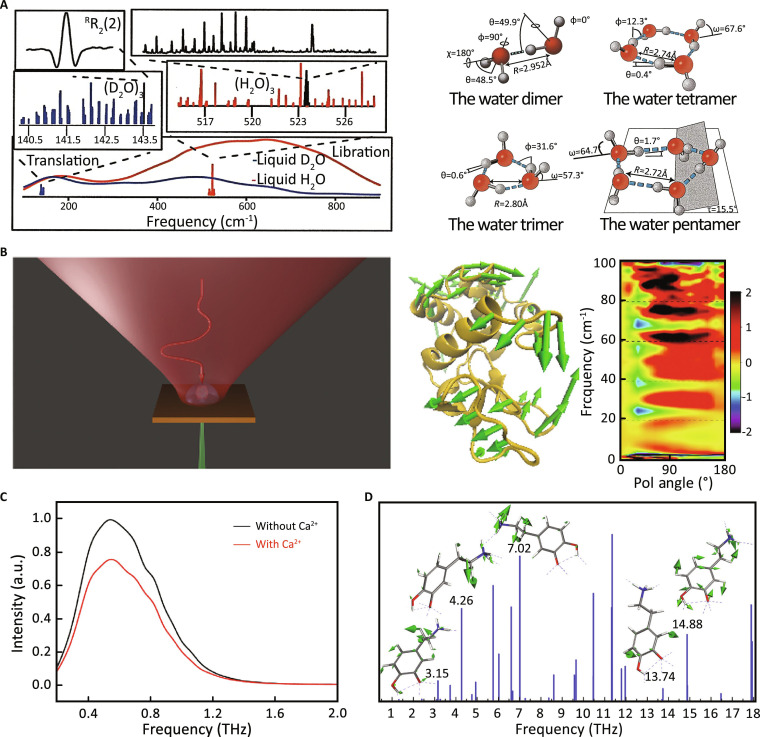
Applications of THz spectroscopy. (A) THz spectroscopy of hydrogen bonding arrangements [155]. Copyright (2001) National Academy of Sciences. (B) Probing directional anisotropy of proteins via THz spectroscopy [158]. (C) THz amplitude spectrum of coagulation factor VIII with and without calcium in water droplets [160]. (D) THz spectrum of dopamine [165].

Firstly, THz spectroscopy has been employed to provide foundational insights into hydration and protein dynamics. For example, Heugen et al. [154] and Keutsch and Saykally [155] demonstrated how THz spectroscopy captures slowed hydrogen-bond rearrangements in hydration shells and cooperative hydrogen bonding in water clusters (Fig. 8A). Penkov [156,157] used THz-TDS to explore distant hydrated regions and protein dielectric properties, highlighting their role in protein function. Moreover, KITA spectroscopy captured fast solvent–protein coupling in protein folding [108], while polarization-sensitive studies revealed directional anisotropy in protein bands (Fig. 8B) [158]. Importantly, Nibali et al. [159] demonstrated both acoustic- and optic-like vibrational modes in protein MD simulations, reflecting the complexity of THz-active motions. These advances firmly establish THz spectroscopy as a key tool to investigate ultrafast collective dynamics across biomolecular systems.

Secondly, the integration of THz spectroscopy into advanced platforms such as microfluidic sensors and theoretical nanoantenna systems has markedly expanded its applications in biological studies. For instance, early investigations by Soussi and Chalopin [160] revealed that even chemically similar lipid bilayers, such as dipalmitoylphosphatidylcholine (DPPC) and 1-palmitoyl-2-oleoylphosphatidylcholine (POPC), display distinct THz spectra due to dipole orientation differences. Subsequently, Tang et al. [161] used a microfluidic THz system for Huntington’s gene mutation detection. Tang et al. [162] developed the droplet THz method to probe hydrated domains in coagulation factor VIII, uncovering Ca^2+^-dependent interdomain motions (Fig. 8C). Tang et al. [161] and Yang et al. [163] used microfluidic THz systems for protein solution analysis. In parallel, Elayan et al. [164] envisioned THz nanoantenna emitters dynamically modulating protein conformation within biochannels by tuning THz frequency and intensity. More recently, Wu et al. [106] employed air plasma THz-TDS with DFT/ONIOM simulations to study l-Glu and its receptor NMDAR, highlighting water-mediated vibrational regulation.

Thirdly, THz spectroscopy has been utilized to quantitatively characterize biomolecules and to identify their spectral fingerprints associated with different physiological states. For instance, Chen et al. [165] demonstrated that THz spectroscopy could differentiate chiral isomers of tartaric acid, highlighting its sensitivity to stereochemical configurations. Morales-Hernández et al. [27] subsequently applied THz–calorimetric correlation analysis to investigate carbohydrate hydration dynamics. Zhu et al. [166] combined THz spectroscopy with molecular docking simulations to analyze dopamine, revealing that hydrogen bonding and conformational variations significantly influence THz absorption features (Fig. 8D). Ding et al. [167] further extended these applications to colloidal systems, exploring particle interactions and aggregation behavior. Hou et al. [168] utilized THz-TDS to detect l-arginine in aqueous environments, while Wang et al. [169] compared crystalline, hydrated, and free forms of l-arginine, linking spectral differences to hydration status. Most recently, Xue et al. [170] demonstrated that THz-TDS can sensitively detect proteinuria in human urine samples, underscoring its diagnostic potential. Altogether, these studies highlight THz spectroscopy’s ability not only to resolve subtle structural and hydration-dependent differences but also to push toward applications in diagnostics, conformational control, and the fundamental understanding of biophysical processes.

### Imaging

THz imaging provides vital spatial information about samples through various techniques, including transmission imaging, reflection imaging, and tomography. With recent technological advances, both spatial and temporal resolutions have improved dramatically, expanding the capacity of THz imaging from structural visualization to dynamic process monitoring.

**Fig. 9.**
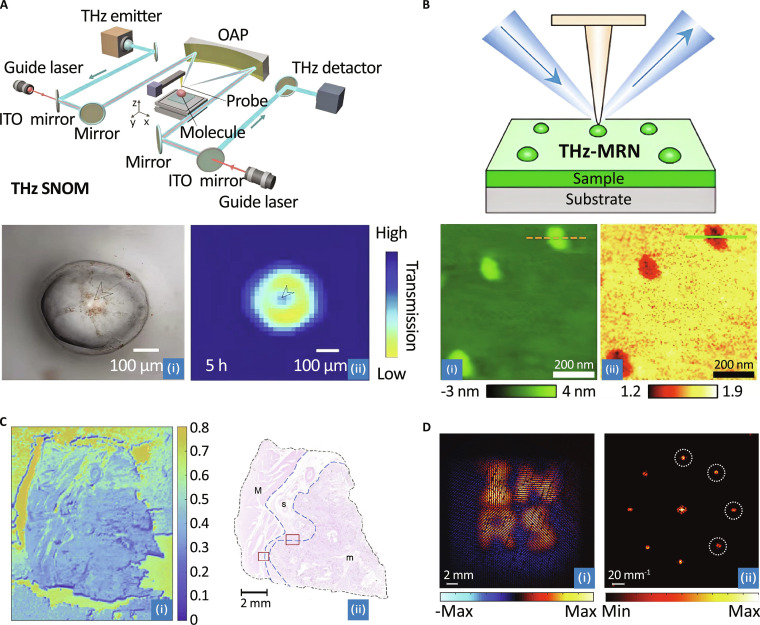
THz applications in imaging. (A) THz scattering-type scanning near-field optical microscopy (s-SNOM) system schematic and its THz imaging of a single watermelon pulp cell [171]. (B) Schematic illustration of THz-MRN and its imaging of single immunoglobulin G (IgG) molecules [172]. (C) THz imaging of gastric tissue layers [174]. (D) THz imaging of optically opaque media [176].

At the microscale and nanoscale, near-field techniques have driven major breakthroughs. THz scanning near-field optical microscopy (THz-SNOM) has surpassed the diffraction limit, enabling submicrometer inspection of biomaterials. Yan et al. [171] comprehensively reviewed the diverse biomedical applications of THz-SNOM and its variants, emphasizing their ability to achieve micrometer- and nanoscale resolution for precise biological inspection (Fig. 9A). The review also addresses current challenges and outlines future development pathways. Extending this paradigm, Yang et al. [172] developed THz morphological reconstruction nanoscopy (THz-MRN), which employs an extended finite dipole model to probe protein layer morphologies with surface and subsurface imaging resolution down to 0.5 nm, enabling characterization at the single-molecule level (Fig. 9B). Most recently, Hu et al. [173] employed THz scattering SNOM to resolve membrane heterogeneity with resolution surpassing atomic force microscopy.

At the tissue level, instrumentation advances have enabled clinical relevance. Shi et al. [174] developed a high-resolution THz imaging system integrating a 3-THz pulsed quantum cascade laser with a 65-nm complementary metal-oxide semiconductor (CMOS)-based detector. This system effectively differentiates cancerous from normal gastric tissue layers by exploiting their distinct THz transmittance properties (Fig. 9C). Meanwhile, Yan et al. [175] summarized the field’s evolution from in vitro applications to in vivo modalities, discussing advances in contrast agents and innovative imaging strategies while providing a strategic roadmap for future progress.

For dynamic biological events, new ultrafast techniques have emerged. For instance, Dong et al. [176] developed a single-shot ultrafast THz photography system. Utilizing spatiotemporal multiplexing of optical probes, this system captures complex ultrafast events within optically opaque media with subpicosecond temporal resolution (Fig. 9D). Collectively, these innovations highlight the expanding role of THz imaging as a high-precision, label-free modality for biophysical and biochemical investigations.

### Sensors and detection

THz-based biosensors have garnered significant attention due to their ability to detect and quantify biomolecules with high sensitivity [177]. These sensors rely on the interaction of THz waves with biological samples, resulting in shifts in resonant frequencies or other electromagnetic properties. In the field of THz biophysics and chemistry, the development of advanced sensors and detection technologies has enabled sensitive, label-free, and high-throughput analysis of biological systems. To systematically summarize these advancements, Table 2 provides a comparative overview of the key THz sensing technologies, their applications, and performance metrics.

**Table 2.** Thematic summary of advances in THz sensors and detection

Research focus	Author and year	Method	Detection target	Main finding
Cellular detection	Shi et al. [178]	Single-shot THz detection (grating pulse)	Cervical cancer cells	Identified cell concentration via absorption peaks
	Zhang et al. [179]	Reflective circular dichroism (Si grating)	Living cancer cells	Detected living cancer cells at 10^4^ cells/ml
	Cao et al. [180]	THz ATR spectroscopy with machine learning	Colorectal cancer cell lines	Achieved classification of cancer cell lines
	Zhang et al. [181]	THz spectroscopy (microdroplet)	Various cell types/states	Enabled automated, high-throughput cell classification
	Yao et al. [182]	All-silicon biosensor (graphene/micro-grooves)	Live cancer cells (aqueous)	Distinguished live cell types in liquid at 5 × 10^4^ cells/ml
Biosensor design and performance optimization	Niessen et al. [183]	Anisotropy THz microscopy	Biomacromolecules (proteins/RNA)	Resolved molecular binding vibrations with 6× faster speed
	Niu et al. [184]	Metamaterial field-confinement biosensor	SARS-CoV-2 S1 protein	Boosted sensitivity via localized field confinement
	Cho et al. [186]	THz-TDS metamaterial platform	Inclusion bodies (*E. coli*)	Enabled rapid detection of protein aggregates in *E. coli*
	Kidavu and Chaudhary [187]	THz photoacoustic spectroscopy	Volatile organic compounds (VOCs)	Achieved ultralow detection of VOCs via photoacoustics
Noise suppression	Lou et al. [191]	Dual-pulse noise-cancellation metasurface	General electromagnetic sensing	Demonstrated calibration-free, robust sensing via noise cancellation
	Zhang et al. [192]	Surface-wave metasurface	Matter characterization	Achieved simultaneous refractive index and fingerprint sensing
	Wang et al. [193]	Split-ring resonator metasensor (Au NPs)	Specific biomarker (via antibody)	Achieved ultrahigh sensitivity of 674 GHz/RIU
Chemical and material detection	Xu et al. [196]	2D COF nanofilms as absorbers	Pesticides	Detected pesticides with sensitivity and robustness
	Liu et al. [197]	Angle-resolved reflected optical pump–THz probe	High-conductive thermoelectric materials	Tracked ultrafast carrier dynamics with angular resolution
	Chang’s group [198]	Femtosecond optical-pump THz-probe spectroscopy	Charge transport (perovskite films)	Quantified charge transport to improve solar cell efficiency mobility

**Fig. 10.**
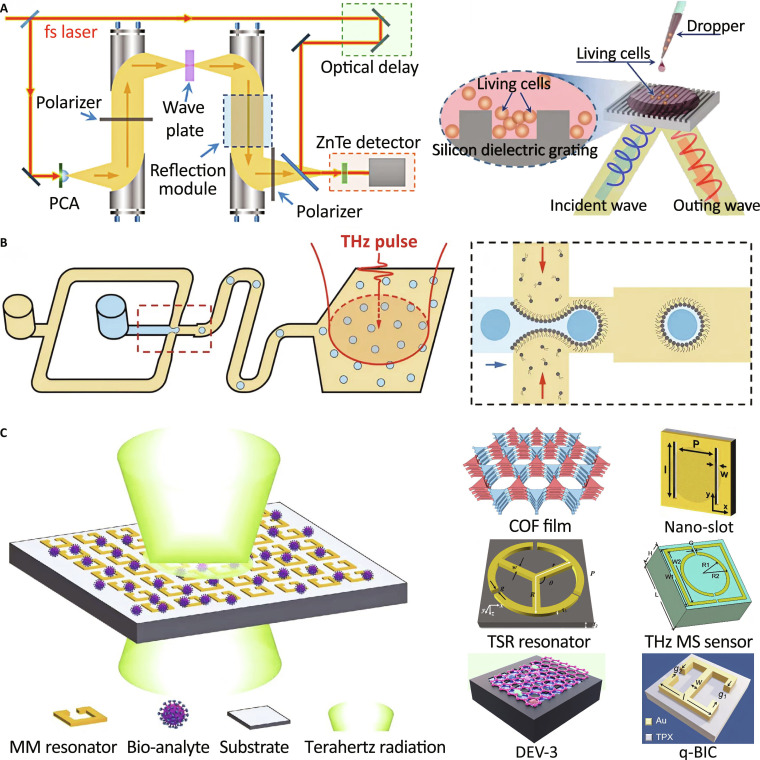
THz applications in sensors and detection. (A) Reflective THz sensing of living cell samples [179]. (B) Integrated THz system for classifying different cell types [181]. (C) THz biosensors [182,184–186,188,189,196].

A primary research thrust focuses on cellular detection. In this context, early work by Shi et al. [178] developed a single-shot THz detection system based on a grating pulse technique, enabling transient detection and identification of characteristic absorption peaks that scale with cervical cancer cell concentration. Zhang et al. [179] proposed a reflective THz time-domain circular dichroism sensing system incorporating a silicon subwavelength grating, achieving highly sensitive detection of living cancer cells at concentrations as low as 10^4^ cells/ml (Fig. 10A). Subsequently, the integration of machine learning techniques further advanced the field. Cao et al. [180] successfully classified colorectal cancer cell lines using THz attenuated total reflection (ATR) spectroscopy. More recently, Zhang et al. [181] developed a label-free system that integrates THz spectroscopy with microdroplet encapsulation, allowing automated high-throughput classification of different cell types and physiological states (Fig. 10B). Extending these advances, Yao et al. [182] developed a reusable, all-silicon THz biosensor integrating graphene and micro-grooved substrates, enabling the distinction of live cancer cell types in aqueous environments with a minimum detection concentration of 5 × 10^4^ cells/ml, assisted by 2D optical cards to enhance accuracy (Fig. 10C).

Another research focus lies in THz biosensor design and performance optimization, where advances in metamaterials and polarization control have substantially enhanced sensitivity and acquisition speed. Niessen et al. [183] introduced polarization-varying anisotropy THz microscopy, capable of resolving binding-induced vibrational changes in biomacromolecules such as proteins and RNA G-quadruplexes, with a 6-fold increase in acquisition speed. Niu et al. [184] demonstrated that analyte localization near resonant gaps in a metamaterial biosensor significantly improves sensitivity through field confinement, as exemplified for the detection of the severe acute respiratory syndrome coronavirus 2 (SARS-CoV-2) S1 protein (Fig. 10C). In 2024, Cao et al. [185] proposed design strategies for THz metamaterial biosensors, emphasizing methods to enhance sensitivity, functionalization, and fabrication, and providing perspectives for future development (Fig. 10C). Cho et al. [186] constructed a THz-TDS metamaterial sensing platform for rapid, label-free detection of inclusion body formation in *E. coli* (Fig. 10C). More recently, Kidavu and Chaudhary [187] applied THz photoacoustic spectroscopy to detect volatile organic compounds, reporting ultralow detection limits and the first observation of THz-based photoacoustic signals.

As a result of design and performance optimization in THz biosensors, the incorporation of resonant structures and plasmonic effects has substantially enhanced both sensitivity and selectivity in molecular detection [188,189]. Early reviews, such as Beruete and Jáuregui-López [190], summarized how engineered THz metasurfaces overcome classical THz limitations to enable enhanced thin-film and biological sensing. However, electromagnetic sensing is frequently disturbed by environmental noise. To overcome this limitation, Chang’s group [191] proposed and demonstrated the revolutionary ideas by dividing 2 picosecond-delayed transmission spectra from a single ultrafast laser-controlled metasurface to cancel out the noise and achieve calibration-free, high-precision, and robust sensing. They further extended this concept to achieve synchronously quantitative refractive sensing and qualitative fingerprint recognizing matter, by exciting and propagating THz surface wave over a long range to accumulate phase difference (refractive variation) and characteristic absorption (fingerprint) [192]. Meanwhile, Wang et al. [193] designed a novel metasensor of split-ring resonator metasurface combining with gold nanoparticles conjugated with the specific antibody, to promisingly achieve an ultrasensitive sensitivity of 674 GHz/RIU. Recent developments have also demonstrated the potential of THz metasurfaces for ultrafast logic operations. For example, Zhang et al. [194] developed a light-driven, ultrafast programmable THz metasurface enabling temporally controlled XNOR, NOR, and OR logic operations, demonstrating miniaturized and multifunctional THz flat optics with picosecond-scale switching. Furthermore, Yuan et al. [195] implemented 3-bit parallel logic operations using a light-driven ultrafast THz metasurface device, demonstrating high-efficiency parallel computing on a single silicon chip within 8 ps.

Beyond biosensing, THz techniques have been extended to diverse chemical and material detection applications. For instance, Xu et al. [196] utilized 2D covalent organic framework (COF) nanofilms as absorbers for sensitive and selective pesticide detection, with excellent mechanical robustness (Fig. 10C). Meanwhile, traditional Hall-effect measurements are limited by contact impedance and unsuitable for dynamic carrier detection, while conventional THz transmission suffers from shallow penetration in highly conductive media. To overcome these limitations, an innovative angle-resolved reflected optical pump–THz probe method was developed, enabling simultaneous tracking of ultrafast carrier dynamics with unprecedented angular resolution for probing anisotropic properties of high-conductive thermoelectric materials [197]. Additionally, THz photons uniquely suppress interband transitions in semiconductors, allowing selective probing of intrinsic free carriers. By developing femtosecond-resolved optical-pump THz-probe spectroscopy, Chang’s group [198] originally quantified charge transport dynamics in perovskite films and groundbreakingly distinguished the individual contributions of electron and ion carriers, revealing that dipolar passivation markedly improves carrier mobility and diffusion length, driving record power-conversion efficiencies in all-perovskite tandem solar cells. The precision of this advanced method greatly validated the solvent-confinement edge-protection strategy, facilitating the design of record-certified module-scale efficiency of perovskite photovoltaics [199]. Collectively, these studies highlight the versatility and sensitivity of THz techniques, spanning applications from chemical detection to material characterization.

## Conclusion

We have provided a comprehensive overview of the mechanisms by which THz radiation interacts with biological and chemical systems, as well as the advances in applications of THz technology in biophysics and chemistry. The central molecular mechanism underlying these interactions lies in the fact that molecules exhibit intrinsic vibrational modes within the generalized THz frequency range, allowing resonant absorption when the radiation frequency matches these vibrations. It allows THz radiation to modulate the dynamic structure and function of biomolecules while also probing molecular motions and enabling high-resolution, noninvasive detection, imaging, and sensing of biological structures and processes at unprecedented scales.

Despite significant progresses in the field of THz biophysics and chemistry, much work remains to be done to further advance its development. A major challenge is the lack of standardized THz radiation parameters and biosafety evaluation protocols, which are essential for ensuring reproducibility and comparability across studies. Equally critical is the urgent need to systematically assess the biocompatibility of THz radiation, particularly its long-term safety at both cellular and organismal levels. Moreover, while theoretical and computational studies have revealed intriguing mechanisms of THz–biomolecule interactions, rigorous experimental validations are indispensable to confirm and refine these theoretical predictions. Addressing these challenged issues will lay a solid foundation for the broader application of THz technologies in biophysics and chemistry. Looking ahead, the interdisciplinary integration of physics, biology, chemistry, and engineering in THz research holds great promise. It may not only deepen our understanding of fundamental scientific questions but also support transformative applications in precision medicine, chemistry, and bioengineering.
